# Causes and Correlates of Calf Mortality in Captive Asian Elephants (*Elephas maximus*)

**DOI:** 10.1371/journal.pone.0032335

**Published:** 2012-03-01

**Authors:** Khyne U. Mar, Mirkka Lahdenperä, Virpi Lummaa

**Affiliations:** 1 Department of Animal and Plant Sciences, University of Sheffield, Sheffield, United Kingdom; 2 Section of Ecology, Department of Biology, University of Turku, Turku, Finland; 3 Wissenschaftskolleg zu Berlin, Institute for Advanced Study, Berlin, Germany; University of Manitoba, Canada

## Abstract

Juvenile mortality is a key factor influencing population growth rate in density-independent, predation-free, well-managed captive populations. Currently at least a quarter of all Asian elephants live in captivity, but both the wild and captive populations are unsustainable with the present fertility and calf mortality rates. Despite the need for detailed data on calf mortality to manage effectively populations and to minimize the need for capture from the wild, very little is known of the causes and correlates of calf mortality in Asian elephants. Here we use the world's largest multigenerational demographic dataset on a semi-captive population of Asian elephants compiled from timber camps in Myanmar to investigate the survival of calves (*n* = 1020) to age five born to captive-born mothers (*n* = 391) between 1960 and 1999. Mortality risk varied significantly across different ages and was higher for males at any age. Maternal reproductive history was associated with large differences in both stillbirth and liveborn mortality risk: first-time mothers had a higher risk of calf loss as did mothers producing another calf soon (<3.7 years) after a previous birth, and when giving birth at older age. Stillbirth (4%) and pre-weaning mortality (25.6%) were considerably lower than those reported for zoo elephants and used in published population viability analyses. A large proportion of deaths were caused by accidents and lack of maternal milk/calf weakness which both might be partly preventable by supplementary feeding of mothers and calves and work reduction of high-risk mothers. Our results on Myanmar timber elephants with an extensive keeping system provide an important comparison to compromised survivorship reported in zoo elephants. They have implications for improving captive working elephant management systems in range countries and for refining population viability analyses with realistic parameter values in order to predict future population size of the Asian elephant.

## Introduction

Life-history analyses of captive animal populations are of both scientific and practical importance for species such as the Asian elephant (*Elephas maximus*) with a significant proportion of the world's population now living in captivity. Studies on the mortality and fertility rates of such populations contribute to our knowledge of how management practices affect population sustainability. In many species conditions in captivity such as stable food availability, medical interventions, improved hygiene and lack of predation are associated with early age at first reproduction, short inter-birth intervals and increased adult longevity in comparison with wild counterparts (reviewed in [1]). However, efforts to achieve self-sustaining captive populations of many species have been hampered by high juvenile mortality rates caused by factors such as obesity, poor adaptation to climate, inbreeding, social stress and incompatible group structure and size [1].

The Asian elephant is classified as Endangered on the International Union for Conservation of Nature (IUCN) Red List of threatened species. In parallel with wild populations, many captive populations are also facing rapid decline and extinction because of current low fertility and high calf mortality rates [2]. A large proportion of remaining Asian elephants live in captivity in range countries (22–30% or 14500–16000 individuals [3]–[4]). Despite the need for detailed data on calf mortality in these remaining captive populations to sustainably manage populations and to minimize the need for capture from the wild, little is known of general trends in Asian elephant calf mortality or its causes and correlates in captive populations. Accurate data on calf mortality is also vital for population viability modeling (e.g. [5]) and is critical to the conservation of remaining often fragmented wild populations as well as captive management. The limited published data on the age-specific levels of mortality from different captive populations are highly variable: while the first year mortality of intensively kept zoo elephants in North America [2] and Europe [6]–[8] is about 30%, first year mortality of timber camp elephants in South India was estimated at 24% for female and 16% for male calves [9]. Such estimates are much higher than the 10–15% first-year mortality reported for wild African elephants (*Loxodonta africana*) of Amboseli, Kenya [10]–[11], but no detailed data has been published of the underlying causes of such differences, or the correlates of varying neonatal or juvenile mortality rates in Asian elephants from the range states.

The paucity of information on the determinants of calf mortality in Asian elephants stems from the rarity of long-term demographic datasets. Studies on captive elephant populations in zoological collections use studbook data but both the sample size and data available on causes of calf mortality in such datasets are often limited. Demographic studies on wild Asian elephants mainly use distance sampling techniques to estimate animal population densities, for example via dung counts or ratios of calves to adults, but no longitudinal, individual-based data exists. Limited information is available for captive Asian elephants [3], [12] kept in South-East Asia under two systems, “extensive” and “intensive” (details in [13]). However, in many of these countries (e.g. Thailand and India), most captive elephants are owned privately as small-holdings and their population size or demography is impossible to monitor due to a lack of systematic registration or governmental influence over movement and changes of ownership of elephants in captivity [3].

The one exception is Myanmar (Burma), home to the largest captive elephant population (*n*>5000) in the world [14]. Half of the elephant population is owned by the government that has recorded and monitored life-history biodata from individuals for over a hundred years. The government-owned captive population has remained stable at 2700 elephants at least from 1999 until 2009 [15], [16]. In Myanmar, elephant draught power has traditionally been extensively used in timber harvesting [17]. Approximately half of the timber elephants used in Myanmar are captive-born, and half are caught from the wild. The elephants live in forest camps, where they are used during the day as riding, transport and draft animals. At night the elephants forage in forests in their family groups unsupervised where they find food and encounter tame and wild conspecifics. Most calves are thought to be sired by wild bulls, and calves born in captivity are cared for by their biological and allomothers, and suckled until lactation no longer supports their demands [18]. Wild-capture of timber elephants was banned in the 1994/95 fiscal year [19] although smaller scale capture of wild elephants (focused on those involved in human-elephant conflict) was resumed again in 2003/04. Given the slow life-history of elephants, calf survival is thus now the major mean of recruitment and determinant of the captive population size. Nevertheless, no studies currently document the causes of calf mortality, compare age-specific mortality rate of males and females, or investigate the effects of maternal age, parity, inter-birth intervals or other maternal attributes on calf success.

Here we use the largest multigenerational demographic dataset on a captive population of Asian elephants, compiled from 260 timber camps in Myanmar over several decades and covering up to four generations of timber elephants. We investigate survival of calves (*n* = 1020) born to captive mothers (*n* = 391), and mortality causes and correlates. Specifically, given the high risk of stillbirths in many captive elephant populations [6], [20], we first examine the overall prevalence of stillbirths, as well as the maternal risk factors associated with the probability of a stillbirth. Second, we document age-specific survival of calves from birth until independence (at age 5) for all liveborn calves produced by captive-born females over their lifespan. Whereas calf mortality is difficult to determine and is likely to be underestimated in field studies on elephants because calves may die and disappear from population before being recorded, records of the Myanmar timber elephants cover all livebirths and most if not all stillbirths. The longitudinal nature of the dataset also allows us to investigate both general between-mother differences in calf mortality, as well as within-mother effects of maternal age, parity and inter-birth intervals, and any calf sex-specific differences therein. We control for geographic variation due to different logging camps, and investigate time trends in calf mortality across our 40-year study period. Finally, detailed cause of death records for nearly all dead calves in our dataset (*n* = 272) allows for documenting for the first time the range and prevalence of causes for death for captive Asian elephant calves in their range country.

## Materials and Methods

### Study population

We used a unique, extensive demographic dataset available on a semi-captive Asian elephant population from Myanmar, covering the full life-history of generations of captive timber elephants. This dataset has been collated from the elephant log-books and annual extraction reports archived and maintained by the Myanmar Timber Enterprise. The traditional elephant log-books are equivalent to the ‘studbooks’ kept in Western zoos. State ownership of thousands of elephants enables recording data of all registered individual elephants from the log-books on: registration number and name; origin (wild-caught or captive-born); date and place of birth; mother's registration number and name; method, year and place of capture (if wild-captured); year or age of taming; dates and identities of all calves born; date of death or last known date alive; and cause of death. The individual elephant log-books are maintained and updated by local veterinarians and regional extraction managers at least bi-monthly in order to check the health condition and ability of each elephant to work. The multiple sources of data recorded by the Myanmar Timber Enterprise (individual elephant log-books coupled with annual extraction reports and end of the year reports from each region) allows effective cross-checking of any apparent errors. Between-individual variation in workload or rest periods is limited by law: all state-owned elephants are subject to the same regulations set by central government for hours of work per week, working days per year, and tonnage to extract per elephant. For example, in 2010 all mature elephants (>17–55) worked 3–5 days a week (depending on weather and forage availability) 5–6 hrs a day (maximum 8 hrs) with a break at noon. All elephants finish their work season by mid-February each year, and work resumes around mid-June depending on the arrival of monsoon. The maximum tonnage of logs allowed to be dragged in a year per elephant was 400 in 2010. The ages of captive-born elephants are exact because precise dates of birth are recorded, and this study concentrates only on the records of captive-born mothers and their offspring in order to have accurate data on maternal age and previous reproductive history, which are incomplete for most wild-captured mothers.

In the wild, elephant calves are raised by their mothers with the help of allomothers and other members of their family unit [21]–[23]. Female calves remain with small numbers of close kin, presumably for their entire lives, while male calves disperse during adolescence (c. 10 years) [24]. In the study population, working females are given rest from mid-pregnancy (11 months into gestation) until the calves reach their first birthday [17]. Mothers are then used for light duties but allowed to nurse the calves on demand. Mahouts or human caretakers do not intervene nor assist in the calving/nursing processes. The calves are generally weaned at the age of four or earlier if they are capable of independent foraging. They are separated from the maternal herd and tamed between ages four and five and then given a mahout, name, a log-book and registration number and trained and used for light work as baggage elephants until age 17. By age 17, they are put into work-force as full working elephants, and retired at age 55 [17], [18].

The entire studbook includes 8006 elephants born and/or captured between 1925 and 2000; data from 2000 onwards is not available to us at the time of this study. Because of the lack of data from 2000 onwards, all calves born in 2000 were removed (*n* = 66) from the analyses, given those born in 2000 would have been censored in all analyses under age one. The remaining sample includes records of a total of 1138 calves born to captive-born mothers, of which 45 calves were recorded as stillborn and included in the analyses of factors associated with the risk of stillbirth. Excluding the stillbirths and animals with missing information on sex or dates of birth/death/lost/escapes, 975 liveborn animals born from 1960 to 1999 (F = 484, M = 491) remained for further analyses from 391 captive-born mothers, themselves born during 1941–1990. These elephants come from 32 timber extraction areas within eight regions (out of a total of 14) in Myanmar: Ayeyarwaddy, Bago, Chin, Kachin, Magway, Mandalay, Sagaing and Shan. The percentage of elephants from the initial sample that were included in the analyses (after excluding individuals with missing or erroneous data in different areas, cohorts or maternal backgrounds) is presented in Table 1. The youngest first-time mother in the sample was 5.3 years (mean age at first reproduction: 18.3±4.7 years; median: 17.8 years) and the oldest reproducing female was 53 years. The average inter-birth interval was 5.4±2.7 years (range = 1.8–18.1 years, median = 4.9) and the maximum lifetime number of calves was 10. These life-history patterns mirror those reported for wild Asian elephants with the earliest age at first reproduction of 6–9 years, a mean age of first reproduction of 17–18 years, the mean inter-birth interval of 2.5–4 years, and maximum number of calves as 12 [4].

**Table 1:** Descriptive statistics of the data used in the analyses (stillbirth and liveborn survival models) of Asian elephants illustrating the sample size and data completeness.

Category	Subcategory	*n*	Follow-up (%)
Sex	Male	587	88.1
	Female	550	91.5
Cohort	1960	94	72.3
	1970	260	82.7
	1980	420	93.1
	1990	364	95.1
Interbirth Interval	Short	167	90.4
	Medium	371	93.8
	Long	173	95.4
	First born/Missing	427	83.4
Birth order	First born	404	84.2
	Later born	734	92.6
Maternal age	<15	61	73.8
	15–24	499	87.2
	25–34	400	93.0
	35–44	155	93.5
	>44	23	100.0
Region	Ayeyarwa	7	85.7
	Bago	75	92.0
	Chin	4	75.0
	Kachin	59	86.4
	Magway	123	95.1
	Mandalay	292	88.4
	Sagaing	473	89.2
	Shan	104	89.4

Initial sample includes all calves born to captive-born mothers before year 2000 *n* = 1138. Follow-up percentage refers to the percentage of elephants in the initial cohort included in the final analyses due to the exclusion of individuals with missing or erroneous data. ‘Short’ interbirth interval refers to cases where the previous birth-interval length was <3.7 years (25% quartile), ‘medium’ if it was 3.7–6.6 years, ‘long’ if it was >6.6 years (75% quartile) and ‘firstborn’ if the calf was first to its mother. Note that logging area (*n* = 32) instead of region (*n* = 8) was used in the statistical models, but values for regions are shown here for simplicity. Firstborn/Missing-category for interbirth interval length includes all firstborns (*n* = 404) and 23 laterborns with missing birth-interval information.

### Statistical analyses

We used a generalized linear mixed model (GLMM) to investigate the stillbirth probability and a discrete time survival analysis (also known as event history analysis) to investigate the calf mortality from birth to age of five. The discrete time survival analysis allows a detailed analysis of the effects of time-dependent variables on the calf's probability of dying over discrete time intervals, while controlling for repeated terms of the mother identity and area [25]. This approach also benefits from including data for some ages also for those individuals that have not been followed until the end of the study period or have not yet reached all the ages investigated in this study (censored), thus avoiding biasing the sample towards those dying young or those with complete records only.

We investigated the effects of calf age (discrete time model), sex, birth-order (1^st^ borns vs. later borns), previous inter-birth interval (short, medium, long as defined below, or firstborn with no interval), birth cohort (decade of birth), mother's age at the birth of the calf as a continuous variable and the repeated measures of the mother and living area (mother's area, *n* = 32). We also included quadratic functions of maternal age in the models. The inter-birth interval preceding the birth of the focal individual was grouped into four classes based on the distribution of interbirth intervals in the population: ‘short’ if the previous birth-interval length was <3.7 years (25% quartile), ‘medium’ if it was 3.7–6.6 years, ‘long’ if it was >6.6 years (75% quartile) and ‘firstborn or missing’ if the calf was first to its mother (most calves in this category) or a laterborn with missing information on birth interval length (*n* = 23). We tested interactions between the fixed variables especially with calf age (discrete time model) to investigate whether any effects change with calf age. The data were restricted to only single-born calves, because of the poor survival prospects and low number of twin births (*n* = 8). Descriptive statistics and follow-up percentages (illustrating data quality) with regard to the variables included in the analyses are given in Table 1 (*n* = 1138, i.e. including 45 stillbirths and animals with missing information on sex or dates of birth/death/lost/escapes that were subsequently excluded from the analysis of livebirths). All analyses were conducted using SAS (SAS Institute, release 9.2, 2002–2008). Significance levels were set at α = 0.05. All biologically interesting interactions were tested, but omitted if not statistically significant. In all analyses, selection of significant terms retained in the best final models was determined using a backward elimination approach.

#### (i) Stillbirth probability

Stillbirth was defined as the birth of a lifeless fetus. The mother's probability of having a stillbirth was investigated using a generalized linear mixed effects model (GLMM) fitted to a binomial error structure with logit link function. The effects of linear and quadratic functions of maternal age, calf sex, birth-order and birth cohort were investigated on stillbirth mortality risk, fitted as fixed terms in the model. Because of the low number of stillbirths, it was not possible to simultaneously estimate the effects of birth intervals and birth-order. Inclusion of only birth-order in our final model was thus chosen, because analyzing both variables separately suggested that birth-order may be more important of the two. Non-independence of stillbirth probabilities of calves from the same mother were accounted by including the mother-id as a repeated term. Mother's area was included as a repeated term to control for similar living conditions of calves from the same area. The analysis was conducted on 1020 calves, of which 45 were stillbirths and 975 live-births.

#### (ii) Calf mortality

We first documented the overall survival pattern (survival distribution function) of calves from birth to age of 18 (average age at first reproduction for females and enrollment into work-force as full working elephant). We then investigated calf mortality from birth (all live-births) to five years using discrete time survival analysis. This analysis allowed us to estimate the calf's risk of dying in each year from birth while investigating the effects of fixed and repeated terms. For each year from birth to 5 years (5 time intervals for each calf; 0–1, 1–2, 2–3, 3–4, 4–5 years), the survival of each calf was coded as survived versus died during the observation year (1/0) or missing (when already dead or disappeared). Following restrictions (see above), this analysis was carried out on 4121 data points (*n* = 975 calves, *n* = 378 mothers).

The analysis investigated the effects of linear and quadratic terms (non-linear effects) of calf age and mother age, previous birth-interval length, calf sex, birth cohort and birth-order and the repeated terms of mother and area. The assessment of interactions between a fixed variable and calf age provides an indication of whether calves are more likely to die at a given age in relation to the variable and thus, whether the effect changes with calf age. Therefore, all interactions with calf age were first included in the model but removed if they did not reach statistical significance level (*P* = 0.05) using the backward elimination approach. We used GLMM with binomial error structure and logit link function in GENMOD procedure of SAS.

#### (iii) Death causes for calves under age 5

The cause of death was recorded for all but 3 liveborn calves out of the total of 227 individuals that died before five years. We classified the death causes into six groups: accidents (including falls, attacks by wild-elephant, tiger or older sibling, drowning, snake bite, strangulation on chains, choking, jammed between trees, head injury, food poisoning); diseases (including unspecified infectious diseases, anthrax, hemorrhagic septicemia, pneumonia, various parasitic infestations and gastrointestinal complications such as constipation, diarrhea, enteritis, colic and bloat); mother agalactia (lack of milk after parturition) and related general weakness; heat stroke; taming-related injury; and others including unknown. We then investigated the distribution and generality of the causes of death for calves below age five.

## Results

### (i) Stillbirth probability

**Figure 1:**
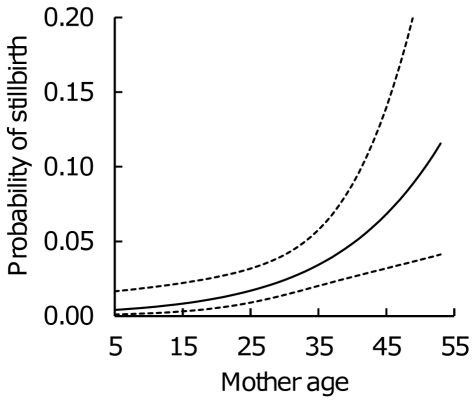
The probability of stillbirth according to maternal age at the birth of calf in Asian elephants. The probability of stillbirth increased significantly with maternal age especially after age 35 (n = 1020 births). Figure shows predicted values and 95% confidence intervals from the model.

Overall, 4% (45/1020) of all calves were stillborn. The probability of stillbirth increased significantly with maternal age (estimate ± S.E. 0.073±0.024, χ^2^_1_ = 5.55, *P* = 0.019; maternal age^2^ χ^2^_1_ = 0.15, *P* = 0.70) Fig. 1A) reaching 9.5% at age 50. Older mothers (≥35 years old) had 3.43 (CIs: 1.08, 10.85) times higher risk of having a stillborn calf than younger mothers in their twenties. Stillbirth probability dropped from being 11.3% (CIs: 7.4, 16.9) for firstborn calves to only 1.8% (CIs: 1.0, 3.4) for laterborn calves, and firstborn calves thus were 6.83 (CIs: 2.86, 16.33) times more likely to be stillborn than subsequent calves of the same mother (χ^2^_1_ = 14,61, *P* = 0.0001). There was no significant difference in stillbirths between males and females (5.1% (CIs: 3.5, 7.5) vs. 4.1% (CIs: 2.6, 6.5), respectively; *n* male = 517, *n* female = 503 including 26 stillborn males and 19 females; χ^2^_1_ = 0.55, *P* = 0.46). Finally, stillbirth probability appeared slightly higher among calves born in 1990s as compared to the other birth cohorts (1960: 3.8% (CIs: 1.1, 12.2), 1970: 3.2% (CIs: 1.3, 7.2), 1980: 3.4% (CIs: 1.9, 6.2), 1990: 7.1% (CIs: 4.6, 10.9)) but the differences between birth cohorts were not statistically significant (χ^2^_3_ = 4.08, *P* = 0.25).

### (ii) Calf mortality

**Figure 2:**
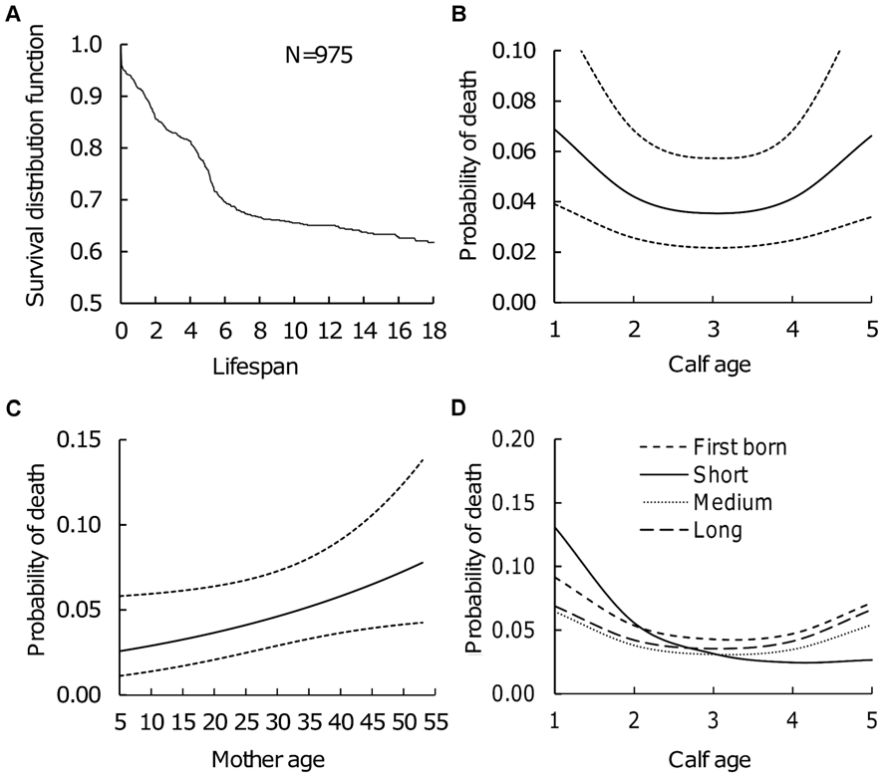
Overall survival distribution function of Asian elephants from birth to age 18 (A) and correlates of mortality of liveborn calves from birth to age of 5 (B–D) (**Table 3**). The mortality risk was highest during the first two years of calf life, and rose again between ages four and five (B). Calves born to older mothers had an increased probability of death irrespective of their own age (C) and short inter-birth interval put calves to higher risk of dying during their first two years of life (previous birth interval and calf age interaction, D). The sample size to which the discrete time survival analysis (B–D) was based was 975 calves with total of 4121 observations and correlated terms also included calf sex, birth order, birth cohort and repeated terms of the same mother and living area (mother id and mother area, see Methods and Table 3). Each year of calf age (1–5) illustrates the probability of dying within the previous full year (B,D, see Methods). The classification of inter-birth interval to first born, short, medium and long was based on the distribution of the birth interval length in the population and to 25% and 75% quartiles (D, see Methods). Figures show predicted values and 95% confidence intervals (B,C) from the model.

Generally, the mortality risk of liveborn calves from birth until age 18 was highest during the first two years of life, followed by another rise in mortality risk between ages four and five when the calves are weaned and their training begins (Fig. 2A). Given the mortality risk quickly leveled off after age five (Fig. 2B), in the following we concentrate on examining factors associated with the high risk of mortality during the first five years of life.

Of the 975 liveborn calves, 25.6% died before reaching age five. The mortality risk varied significantly across different ages in a curvilinear pattern, rising to 6–7% per year during the first two years of life as well as between ages four and five (Fig. 2B, Tables 2 and 3).

**Table 2:** Mortality of Asian elephant calves by each age group from birth to age of 5 years.

Calf age	n died	n survived	% died
0–1	80	895	7.06
1–2	56	824	6.36
2–3	28	775	3.49
3–4	14	735	1.87
4–5	49	665	6.86
total(0–5)	227	665	25.6

n = 975, includes all calves used in the time-discrete survival model.

**Table 3:** Discrete time survival model of the Asian elephant calf mortality risk from birth to 5 years (*n* = 975 calves with 4121 observations (227 deaths)).

Term	Category	Estimate±SE	Statistic (χ^2^_df_)	*P* value	OR(CIs)
Calf age		−0.69±0.19	29.24_1_	<0.0001	
Calf age^2^		0.17±0.046	12.25_1_	0.0005	
Sex			3.85_1_	0.050	
	female	0.00±0.00			1.00
	male	0.26±0.13			1.30(1.00,1.68)
Birth-order			5.34_1_	0.021	
	1^st^ born	1.48±1.00			4.40(0.62,31.3)
	later born	0.00±0.00			1.00
Mother's age		0.024±0.012	3.74_1_	0.053	
Birth cohort			9.71_3_	0.021	
	1960	0.69±0.40			1.99(0.91,4.33)
	1970	0.16±0.29			1.18(0.66,2.09)
	1980	−0.46±0.23			0.63(0.40,0.99)
	1990	0.00±0.00			1.00
Prev. birth interval			10.16_3_	0.017	
	short	0.71±0.35			2.03(1.03,4.02)
	medium	−0.07±0.30			0.93(0.51,1.68)
	1^st^ born	−1.17±1.04			0.31(0.041,2.36)
	long	0.00±0.00			1.00
Age*Prev.birth interval		Fig. 2d	9.06_3_	0.029	
Age*Birth cohort			13.61_3_	0.0035	
Constant		−3.23±0.51			
Mother's age^2^		−0.0013±0.0011	1.59_1_	0.21	
Sex*Birth-order			1.13_1_	0.29	
Age*Mother's age		−0.011±0.0070	2.49_1_	0.11	
Age*Sex		−0.044±0.091	0.23_1_	0.63	
Age*Birth-order		20.092±0.14	2.13_1_	0.14	
Mum age*Birth order		0.036±0.024	2.24_1_	0.13	

Estimates with standard errors (S.E.) (positive estimate reflects increasing mortality risk) are provided for all variables (except interactions with several categories) and odds ratios (OR) with 95% confidence intervals (CIs) for categorical variables. Terms retained in the final model are shown above the constant, whereas examples of those that were rejected from the final model are shown below it. Mother's identity and area were fitted as repeated terms.

Calves born to older mothers tended to have an increased mortality risk at all ages from birth to five years (Table 3; Fig. 2C). Calf mortality risk increased with maternal age so that for calves born to mothers aged 50 or above, the mortality risk was already 3.5-fold (CIs: 0.82, 15.14) compared to those born to mothers in their twenties. For example, calves born to mothers in their twenties had a 81.1% probability of survival to age 5 whereas calves born to mothers ≥50 years of age had on average only a 67.7% probability of survival to age 5.

Producing calves at short inter-birth intervals resulted in increased calf mortality risk, but this effect varied significantly according to the age of the calf (Table 3), so that the differences were largest during the first year of life. Calves born to mothers who had produced their previous calf less than 3.7 years earlier (25% quartile of the birth interval distribution) had a 1.5-fold (CIs: 0.87, 2.74) higher mortality risk during their first year (13.1% (CIs: 8.0, 20.6)) as compared to calves born with long interbirth intervals (6.9% (CIs: 3.9, 11.8)) (Fig. 2D). Such differences largely disappeared with the age of the calf.

Firstborn calves had 4.4 (CI: 0.62, 31.3) times higher mortality risk than did later born calves from the same mothers, and such differences remained from birth until age five (no significant age x birth-order interaction, Table 3). This translated to firstborn calves having only a 51.9% survival probability to age 5, whereas laterborn calves had a 77.7% probability to survive to age 5. There was some tendency for particularly high mortality risk of firstborn calves produced by relatively old first-time mothers, but the interaction between maternal age and parity did not reach statistical significance (Table 3).

Male calves had 1.3 (CI: 1.00, 1.68) times higher mortality risk than female calves across all ages since birth until age five (Table 3). As a consequence, male calves had a 69.2% probability to survive to age 5, whereas female calves had a 75.0% probability to survive to age 5. The calf mortality risk also varied significantly across the study period with the best first year survival in 1980s (93.2% (CI: 88.3, 96.2)) and worst first year survival in 1960s (84.2% (CI: 73.5, 91.8)), but no consistent time pattern emerged across all age-groups (Table 3).

### (iii) Death causes for calves under age 5

The main cause of death for calves under five was accidents (42.4%) which were distributed across all ages (Table 4). One in four (26.3%) died, usually during the first year of life, from mother agalactia (lack of milk) and/or general weakness of the newborn. Various diseases representing mainly gastrointestinal complications accounted for 22.8% of all deaths. Other causes such as taming-related injuries were relatively rare (4.5%). There were no clear overall sex-specific differences in any of the causes of death (Table 4).

**Table 4:** Death causes of Asian elephant calves dying before age 5 by sex and age group (*n* = 224).

Death cause	Females by age group (*n*)					Males by age group (*n*)				
	0–1	1–4	4–5	total	%	0–1	1–4	4–5	total	%
Accidents	11	26	5	42	43.3	19	27	7	53	41.7
General weakness and mother agalactia	20	7	1	28	28.9	20	4	7	31	24.4
Diseases	2	9	8	19	19.6	4	20	8	32	25.2
Taming-related injury	0	0	5	5	5.1	0	1	4	5	3.9
Heat stroke	0	0	1	1	1.0	1	2	0	3	2.4
Others	1	1	0	2	2.1	1	1	1	3	2.4

## Discussion

The worldwide populations of both wild and many captive Asian elephants will continue to be in danger of disappearing unless their current fertility and calf mortality rates improve [2]. A quarter of all remaining Asian elephants live in captivity in range countries in Asia and a further one thousand in zoos around the world [13]. The poor sustainability of many captive elephant populations is particularly constrained by a high calf mortality rate. We used the largest multigenerational demographic dataset in the world on a captive population of Asian elephants compiled from timber camps in Myanmar to investigate calf mortality patterns in detail for the first time. This is to our knowledge the only existing longitudinal dataset on captive Asian elephant from their range countries, and allowed us to explore not only general calf-specific differences in mortality, but also both between-mother and within-mother risk factors and temporal and geographic variation therein, as well as specific causes of death. Our results on extensively managed Myanmar timber elephants provide an important comparison to compromised survivorship reported in zoo elephants. They have implications for improving the management of captive working elephants in Asia as well as for refining population viability analyses with realistic parameter values.

Our finding that the general levels of stillbirth and pre-weaning mortality among Myanmar working elephants were considerably lower than those reported for zoo elephants is of interest. The recent progress in our understanding of physiology [26]–[27] and assisted reproductive technology in elephants [28]–[29] have lead to enhanced birth rates in zoo elephants [20]. However, such success is mitigated by the high calf mortality rate in zoo-born elephants (details in [20], [30]–[31]. While zoo elephants experience high mortality due to sub-optimal climate, social group and feeding regime combined with prevalent reproductive problems [6], [8], [32]–[33], the wellbeing of captive elephants in timber camps of Southeast Asia living in an environment with more appropriate climate, social group and foraging/feeding regime could be compromised by husbandry and management [3], [34] and work-related stress [18]. Unfortunately little data exist to compare our mortality figures with those of wild Asian elephants [4]. Our results on the working timber elephants thus add a significant comparison point for the zoo data, because although data exist on the reproductive patterns of other intensively maintained captive Asian elephants of South India and Sri Lanka (e.g. [9], [30]), limited information has previously been available on the proximate causes of neonatal mortality in captive Asian elephants and virtually no previous study exists that follows the reproductive events of the same females throughout their lives.

One striking difference between zoo populations and working elephants in Myanmar is the stillbirth rate. During the past two decades, the reports on stillbirth rate in zoo-born Asian elephants have ranged between 16% [6]–[7] and 35% [20] of total births. In contrast, stillbirths have been thought to be rare in extensively kept and wild–living elephants [6], [30] but few previous data have been published on this. Among the timber elephants in Myanmar, only 4% of both all male and female births resulted in stillbirths across the 40-year study period. Such a large difference as compared to zoo elephants is unlikely to be caused by undetected pregnancies in Myanmar because mahouts check their elephant health on a daily basis [17]. Mortality of liveborn calves was also lower among the working elephants than in zoo populations: 7% of calves died under one year of age and altogether a quarter before the age of five. Our data suggests a U-shaped pattern of survival during early years, with a high risk of mortality during the first two years of age and another peak between ages four and five years when calves are weaned and taming begins; similar double pulse in mortality of young elephants is also evident for a number of wild African elephant populations [35]. By comparison, the most recently estimated first year mortality is 30% for Asian elephants of both North American and European zoos (e.g. [2], [8], which is about 6 times higher than our figure. No detailed data exists on wild Asian elephants, but in the Amboseli African elephants, 11% of females and 15% of males died in their first two years of life and altogether 19% of known calves died by the age of five [11]. It appears that the high calf mortality rates reported for zoo populations may be specific to populations kept under such conditions, and elephants kept under extensive keeping system in range countries as well as possibly also wild populations experience far lower mortality rates [see also 8], [30]. Although the results on Myanmar timber elephants are not directly relevant for example to zoo populations, a comparison between the different keeping systems might help to determine improvements that are required for the welfare of captive Asian elephants across the world [30].

Our results offer possibilities to improve the management and sustainability of the large captive working elephant populations in Asia. Given that in the wild the Asian elephant is listed as endangered and the captive populations form a large proportion of the worldwide remaining Asian elephants [3]–[4], understanding the factors which influence the ability of females to reproduce successfully both in natural conditions and in captivity, and finding ways in which to mitigate calf mortality risk, will help to maintain the large demands for captive populations of elephants in many countries without a need to capture elephants from the wild. This is of importance because for example, although many Asian elephant range countries have banned logging, captive working elephants remain important in the forestry industry in Myanmar. Our study identified several mother-related risk factors for increased stillbirth rate and calf mortality. Primiparous mothers (in particular those over the median age of first birth) had over 4-times higher risk of losing their liveborn calf, as compared to their later births, and they also had nearly 7-times higher risk of a stillbirth. Furthermore, calves born to older mothers had an increased risk of stillbirth and postnatal mortality, and short inter-birth interval put calves to higher risk of dying during their first two years of life. The effects reported here of maternal status, age as well as offspring sex on calf survival in Asian elephants generally support those reported previously for shorter-lived mammals, in particularly in ungulates [36]–[37]. That the results from Asian elephants confirm those for other large herbivores is of interest because mammals with extended longevity are under-represented in the current studies on evolutionary ecology and most theories are not tested with species living more than ∼15 years. It is possible that repeated pregnancies without systematic provisioning in female timber elephants, coupled with lactational demands from previous calves could cause malnutrition in older Myanmar elephants that rely solely on natural foraging. These under-nourished older females may fail to maintain full-term pregnancy or to bear a healthy foetus to term, and may also struggle to meet the nutritional demands of live born calves.

It thus appears that maternal health and survival of calves could be improved by systematic supplementation and or along with reduced work-load of females in the risk groups. In order to enhance survival of calves, it might be necessary to ensure that older females and those with short birth-intervals take a long enough rest to replenish their body reserves after producing and suckling an offspring. It may also be advisable to supplement primiparous pregnant females and/or reduce their workload to avoid excess calf mortality. Furthermore, it is likely that social networks with older herd members are particularly important for the breeding success of first-time elephant mothers, given that in the wild, calves are typically raised by their mothers with the help of other related females [21]–[23]. In timber camps, elephants are grouped into units comprised of 6–7 animals. Calves are allowed to stay with and be nursed by their mothers around the clock until weaning. Although not all adult females live with their siblings or mothers in the same area/camp, the support of kin could be crucial at least in early reproductive attempts. Further research is needed on the effects of relationships between related and unrelated females on raising calves [11] to understand how physical and social attributes impact reproduction and offspring survival in Asian elephants in captivity.

The largest fractions of deaths were caused by accidents and by maternal agalactia or innate calf weakness. These latter causes might be partly preventable by supplementary feeding and/or work reduction of the high risk first time or ageing mothers. Over 40% of all liveborn calves died in accidents. They were often linked with a calf whose mother did not produce enough milk straying away from the mother in search of additional food, and might thus be avoidable by improving the nutrition of such calves and their mothers. The main causes for calf-weakness related deaths are inability of mothers to produce adequate milk following pregnancy and nutritional stress, as well as parasite infections of calves [11], [38]. Males are known to be more susceptibility to parasitism than females [39], and they may also die more often from various diseases between ages one and four (Table 4). Overall, male calves had higher mortality rate at all ages as compared to females, likely caused by their higher infection rate as well as the fact that they were more likely to encounter accidents when searching for supplementary food as male calves are larger, require more energy to sustain adequate growth and are more expensive for mothers to produce [40]. Studies on Amboseli African elephants show that mother-calf distance increases with calf age and this occurs more rapidly if the mothers do not produce enough milk, and male calves tend to break the mother-offspring relationship earlier than female calves [11], [40]. A third of all accidents in our study occurred during the summer months, when water and fodder are not as abundant and as nutritious as in other seasons and mothers are struggling to nurse their calves, despite not working and being able to forage ad-libitum along with their calves.

These results have implications for refining population viability analyses with realistic parameter values in order to understand the sustainability of captive elephant populations. Several attempts have been made recently to estimate the future population of the Asian elephant. For example, Leimgruber *et al.* [5] applied a Population Viability Analysis (PVA) using estimated demographic rates and population sizes to investigate the demographic link between captive and wild elephant populations in Myanmar, and the past, present and future consequences of continued live capture for remaining wild populations. They concluded that elephants will likely disappear from the wild in Myanmar in 31 years. A major weakness in such analyses has been the lack of detailed life-history data, in addition to disagreements over the wild population size and off-take from the wild. Coulson *et al.* [41] concluded that PVAs are good at predicting population dynamics and extinction probabilities only when extensive and reliable demographic data are available. The assumption of Myanmar elephant mortality schedules in Leimgruber *et al.* [5] was based on scattered information on wild elephant demography from other countries [42]–[43]. Our data show that assumptions such as equal mortality rates for females and males or correspondence of survivorship patterns in wild and captive population, as well as a lack of systematic assessment on the age groups most vulnerable to mortality may be inaccurate and may lead to biased population viability estimates. For example, the calf mortality rate for the captive population of Myanmar by Leimgruber *et al.* [5] corresponds to 34% of calves of both sexes dying by age 5, whereas in our study 25.6% of total liveborn calves died before age five with 1.3 times higher mortality risk among males. Future investigations are needed to project population growth rate for both captive and wild populations, taking into account the effects of female population age structure and parity on their calf mortality rate and sex differences therein. Another factor to consider is that the birth sex-ratios are close to 50∶50, and while males do not contribute to population parameters, they do contribute to the work done in the timber industry. Therefore the differential mortality of males might contribute to a need to capture more males for work, affecting the breeding success of the wild populations even further.
